# Annual Meeting of the Indiana State Dental Society

**Published:** 1896-07

**Authors:** 


### EDITORIAL.

### Annual Meeting of the Indiana State Dental Society.

This body held its regular annual meeting on June 30th and the following two days. The meeting was fairly-well attended, and mainly by the representative members of the State; a goodly addition was made to the membership, which is a hopeful indication. A number of very good papers were read, and well discussed; the discussions were participated in by more than the usual number, relatively, many of the younger members taking part.

The address of the President was of much interest and drew out a very interesting discussion, a large number taking part. The manner of the appointment of Examining Boards was freely considered, and the almost unanimous conclusion was reached that the appointing power should be in the hands of the State Societies, and, at least, the power to nominate; about twice the number to be appointed should be nominated by the State Societies, and from these the Governor might appoint. The general opinion seemed to prevail, however, that there is no necessity for the action of the Governor of the State at all in this matter.

The subject of granting certificates of qualification to non-graduates by Dental Examining Boards was fully discussed, and the general opinion was that it ought not to be done, except in very rare cases. And they would be rare, indeed, if Examining Boards did their duty to the full.

The opinion was freely expressed that no one ought to be licensed to practice dentistry without a qualification equal to that required by our best dental colleges. This is the position taken by quite a number of the Examining Boards in the country, and the idea is becoming more and more prevalent.

The subject of "Replanting and Transplanting Teeth" called out some interesting discussion, and while there was no specially new ideas, some of the old points were so rubbed up as to be quite attractive and interesting.

The subject, "Necrosis," was presented in a very interesting paper by Dr. N. W. Hiatt. The paper called out a prolonged discussion, and one of more than ordinary interest.

A number of other subjects were presented and discussed, among which were "Antiseptics," "Dental Nomenclature," "The Dentist," "The Benefit of Dental Meetings," etc.

The meeting fully sustained the reputation of the Indiana State Dental Society; and, indeed, it was said by many that so good a meeting had not been held for years. The fact is that nearly every one took some part—and that always makes a good meeting. May the future always show as good meetings as the one just closed.

